# S100A9 protein is a novel ligand for the receptor CD85j and their interaction is implicated in the NK cell-mediated control of HIV-1 replication

**DOI:** 10.1186/1742-4690-9-S2-P185

**Published:** 2012-09-13

**Authors:** UY Moreno Nieves, V Arnold, JS Cummings, C Didier, A Gilbert, F Barré-Sinoussi, D Scott-Algara

**Affiliations:** 1 Institut Pasteur, Paris, France

## Background

CD85j is a receptor expressed by different cells of the human immune system including Natural Killer (NK) cells. Previous reports have shown that CD85j interacts with several MHC-I molecules, as well as with some viral proteins (Cosman D et al., 1997). We have also demonstrated that CD85j^+^ NK cells efficiently control HIV-1 replication in monocyte-derived dendritic cells (MDDC) in vitro (Scott-Algara D et al., 2008). We hypothesize that the CD85j^+^ NK cell-mediated anti-HIV activity in MDDC is specifically dependent on the interaction between CD85j receptor and unknown non-HLA class I ligand(s). Therefore we focused on the identification CD85j ligand(s) and its(their) implication in the control of HIV-1 infection.

## Methods

To identify the CD85j ligand(s), lysates from MDDC infected or not by HIV-1 were co-immunoprecipitated using CD85j recombinant receptor and analyzed by SELDI-TOF-MS protein chip arrays. The interaction between CD85j and its ligand(s) where then confirmed by ELISA test. Surface expression of the putative ligand(s) was analyzed by flow cytometry. To confirm the implication of the interaction receptor-ligand in the control of HIV replication, NK cells were pre-stimulated with CD85j ligands and co-cultured with HIV-infected MDDC or CD4^+^ T cells, then, intracellular and supernatant p24 were measured.

## Results

We found that the CD85j receptor interacts with the calcium-binding protein S100A9. We further demonstrated that HIV-1 infection of MDDC modulates the expression of S100 proteins at the surface of MDDC. Pre-stimulation of NK cells with S100A9 monomers resulted in an increased control of HIV infection in MDDC and CD4^+^ T cells. Moreover, pre-stimulation of NK cells with S100A9 tetramers resulted in a better and increased control of HIV-1 infection in CD4^+^ T cells.

## Conclusion

Triggering the inhibitory receptor CD85j on NK cells by S100A9 may be implicated in the establishment and/or the regulation of the specific anti-HIV-1 NK cell response.

